# Granulocyte and Monocyte Adsorptive Apheresis Maintenance Therapy Restored the Loss of Response to Anti‐TNF‐Alpha Agents in the Patients With UC: A Case Report

**DOI:** 10.1002/jca.70030

**Published:** 2025-05-02

**Authors:** Nobuhiro Ueno, Yu Kobayashi, Aki Sakatani, Tatsuya Dokoshi, Keitaro Takahashi, Katsuyoshi Ando, Shin Kashima, Kentaro Moriichi, Hiroki Tanabe, Yuki Kamikokura, Mishie Tanino, Mikihiro Fujiya

**Affiliations:** ^1^ Department of General Medicine Asahikawa Medical University Hospital Asahikawa Hokkaido Japan; ^2^ Department of Gastroenterological Sciences Asahikawa Medical University Asahikawa Hokkaido Japan; ^3^ Division of Gastroenterology, Department of Medicine Asahikawa Medical University Asahikawa Hokkaido Japan; ^4^ Department of Diagnostic Pathology Asahikawa Medical University Hospital Asahikawa Hokkaido Japan

**Keywords:** anti‐TNF‐α agents, granulocyte and monocyte adsorptive apheresis (GMA), loss of response, maintenance therapy, ulcerative colitis

## Abstract

Ulcerative colitis (UC) is a chronic inflammatory condition requiring lifelong management, with anti‐tumor necrosis factor α (anti‐TNF‐α) agents often used for refractory cases. However, secondary loss of response (LOR) to these agents, due to anti‐drug antibodies, poses a significant therapeutic challenge. This report describes a case where granulocyte and monocyte adsorptive apheresis (GMA) maintenance therapy successfully restored the efficacy of an anti‐TNF‐α agent in a 26‐year‐old male with active UC experiencing LOR to infliximab. Following GMA induction therapy and continued infliximab administration, clinical symptoms improved, fecal calprotectin levels decreased, and clinical remission was achieved. Long‐term maintenance with GMA enabled sustained clinical remission, with mucosal healing observed one year post‐therapy. This case suggests that GMA maintenance therapy may serve as a novel therapeutic approach for patients with active UC experiencing LOR to anti‐TNF‐α agents. However, further studies are warranted to elucidate the underlying mechanisms and validate its efficacy.

## 1 Introduction

Ulcerative colitis (UC) is an idiopathic, chronic inflammatory disorder requiring lifelong medications, including immunosuppressive therapy [1]. Recent findings indicate that the complex dysregulation of cytokine expression in the intestinal mucosa plays a significant role in the pathology of inflammatory bowel disease (IBD) [2]. Consequently, treatment strategies targeting various cytokines, such as biologics, have been increasingly utilized in clinical practice. Among these, tumor necrosis factor α (TNF‐α) is known to be a key mediator in the pathogenesis of UC, and the clinical efficacy of anti‐TNF‐α agents has been well established [3, 4, 5, 6]. In Japan, three anti‐TNF‐α agents—infliximab, adalimumab, and golimumab—are approved for the treatment of UC. According to Japanese treatment guidelines, biologics including anti‐TNF‐α agents are generally introduced following inadequate response to conventional therapies, such as 5‐aminosalicylic acid (5‐ASA), corticosteroids, and immunomodulators [7]. Although most UC patients can achieve clinical remission through conventional therapies, a subset does not respond to these treatments. It has been reported that approximately 30% of UC patients become steroid‐dependent, experiencing symptom flare‐ups upon attempting to discontinue steroid therapy, while around 20% are resistant to steroids from the outset [8]. In such cases of steroid‐resistant or steroid‐dependent, anti‐TNF‐α agents are recommended to achieve and maintain clinical remission [7]. However, despite the proven efficacy of anti‐TNF‐α agents during induction therapy, their effectiveness often diminishes during maintenance therapy, leading to relapse. The development of anti‐drug antibodies (ADA) against anti‐TNF‐α agents is believed to be a major cause of this secondary loss of response (LOR), posing a significant challenge that can ultimately necessitate treatment discontinuation [9]. Therefore, strategies to counteract secondary LOR to anti‐TNF‐α agents are critically important.

Granulocyte and monocyte adsorptive apheresis (GMA) using the Adacolumn (JIMRO Co., Takasaki, Japan) has emerged as a non‐pharmacological therapeutic strategy and is widely utilized for both induction and maintenance therapy in patients with active UC in Japan. The GMA mechanism utilizes the Adacolumn, which contains cellulose acetate beads that interact with fragment crystallizable gamma receptors on activated leukocytes, enabling the selective adsorption of granulocytes and monocytes from the systemic circulation [10]. Within the Adacolumn, activated granulocytes and monocytes undergo degranulation and apoptosis. The apoptotic neutrophils that re‐enter systemic circulation from the Adacolumn are processed by secondary lymphoid organs and are phagocytosed by CD19(+) B‐cells. These CD19(+) B‐cells differentiate into regulatory B‐cells, which subsequently inhibit activated lymphocytes by increasing the production of immunosuppressive cytokines. These regulatory cells migrate to inflammatory sites, thereby reducing the secretion of pro‐inflammatory cytokines [11, 12, 13]. This mechanism is believed to contribute to the persistence of anti‐inflammatory effects even after the completion of GMA therapy. The clinical efficacy and safety of GMA have been demonstrated in numerous reports involving patients with active UC [14, 15, 16]. Moreover, there has been a growing interest in combining GMA with biologic therapies [17, 18, 19]. Notably, GMA induction therapy has shown efficacy in active UC patients with LOR to anti‐TNF‐α agents [20, 21, 22]. These reports suggest that GMA could serve as a viable therapeutic option for active UC with secondary LOR to anti‐TNF‐α agents, although current evidence primarily addresses its short‐term efficacy, with limited data on long‐term outcomes. This report describes a case in which GMA maintenance therapy restored the efficacy of an anti‐TNF‐α agent and sustained long‐term clinical remission in an active UC patient with LOR to such agent.

## 2 Case Presentation

**FIGURE 1:**
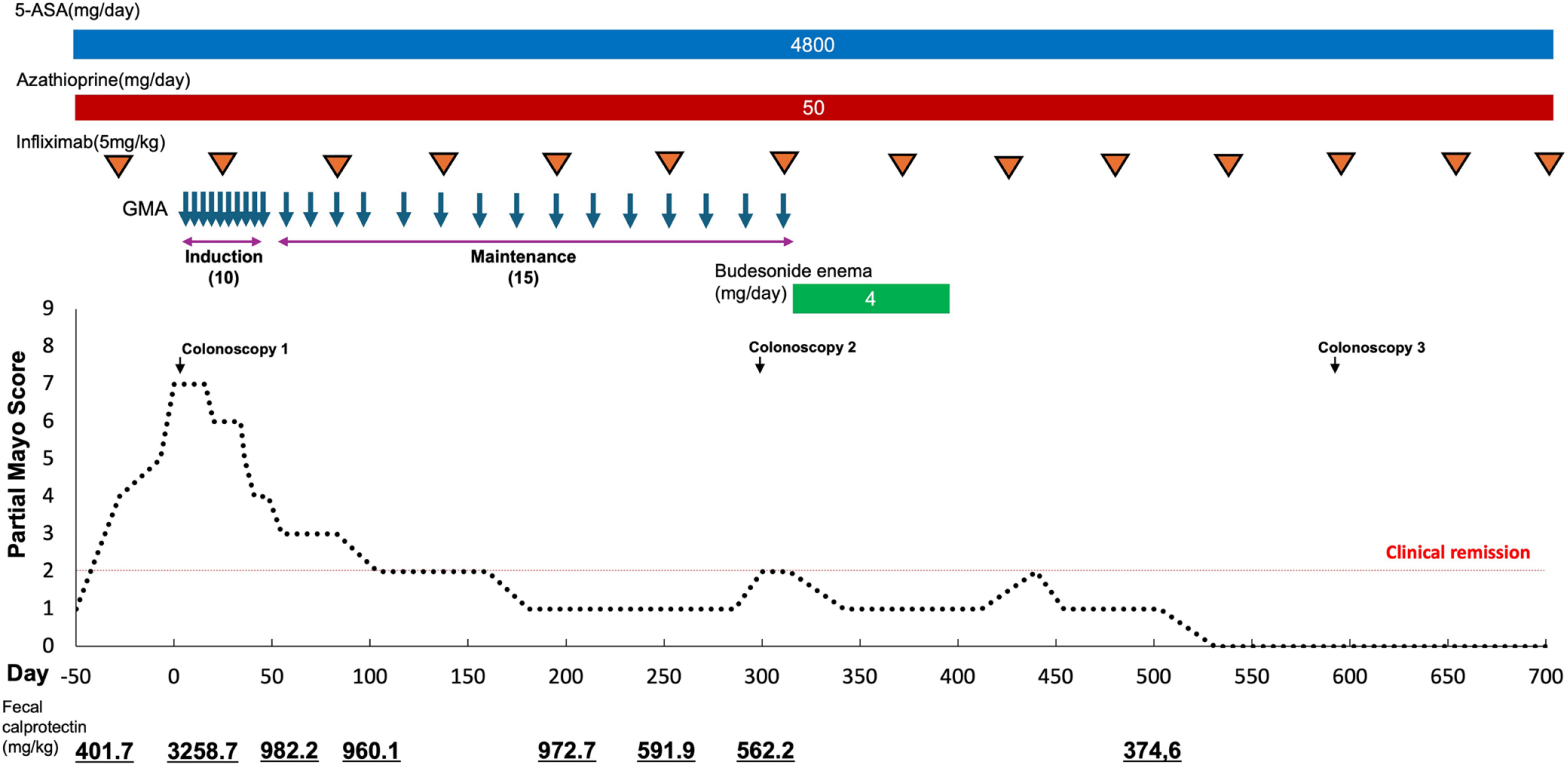
Summarized complete clinical course. 5‐ASA; 5‐aminosalicylic acid.

The complete clinical course is summarized in Figure 1. Clinical activity was evaluated using the partial Mayo score (PMS), and endoscopic activity was assessed using the Mayo endoscopic score (MES) [23]. PMS is composed of three parameters: stool frequency (1–3 points), rectal bleeding (1–3 points), and physician's global assessment (1–3 points), with a total score ranging from 0 to 9. A PMS of 2 or less indicates clinical remission, while a score of 3 or more indicates active disease. MES defines endoscopic remission (mucosal remission) as a score of 1 or less, and active disease as a score of 2 or more. The Montreal classification was used to evaluate the type of disease. The Montreal classification categorizes the types of disease into three groups: E1 (ulcerative proctitis), E2 (left‐sided UC), and E3 (extensive UC), based on endoscopic findings [24]. In addition, pathological activity was assessed using the Geboes score. The Geboes score is a histological grading system used to assess inflammation in UC. This score is semi‐quantitative, structured into six main grades (0–5), with subgrades within each. It evaluates both architectural changes and inflammatory activity. Grades 0–2 generally reflect inactive or chronic inflammation. Grades ≥ 3.1 (presence of neutrophils in epithelium) are considered indicative of active disease. Grade 5 represents the most severe histological damage [25].

**FIGURE 2:**
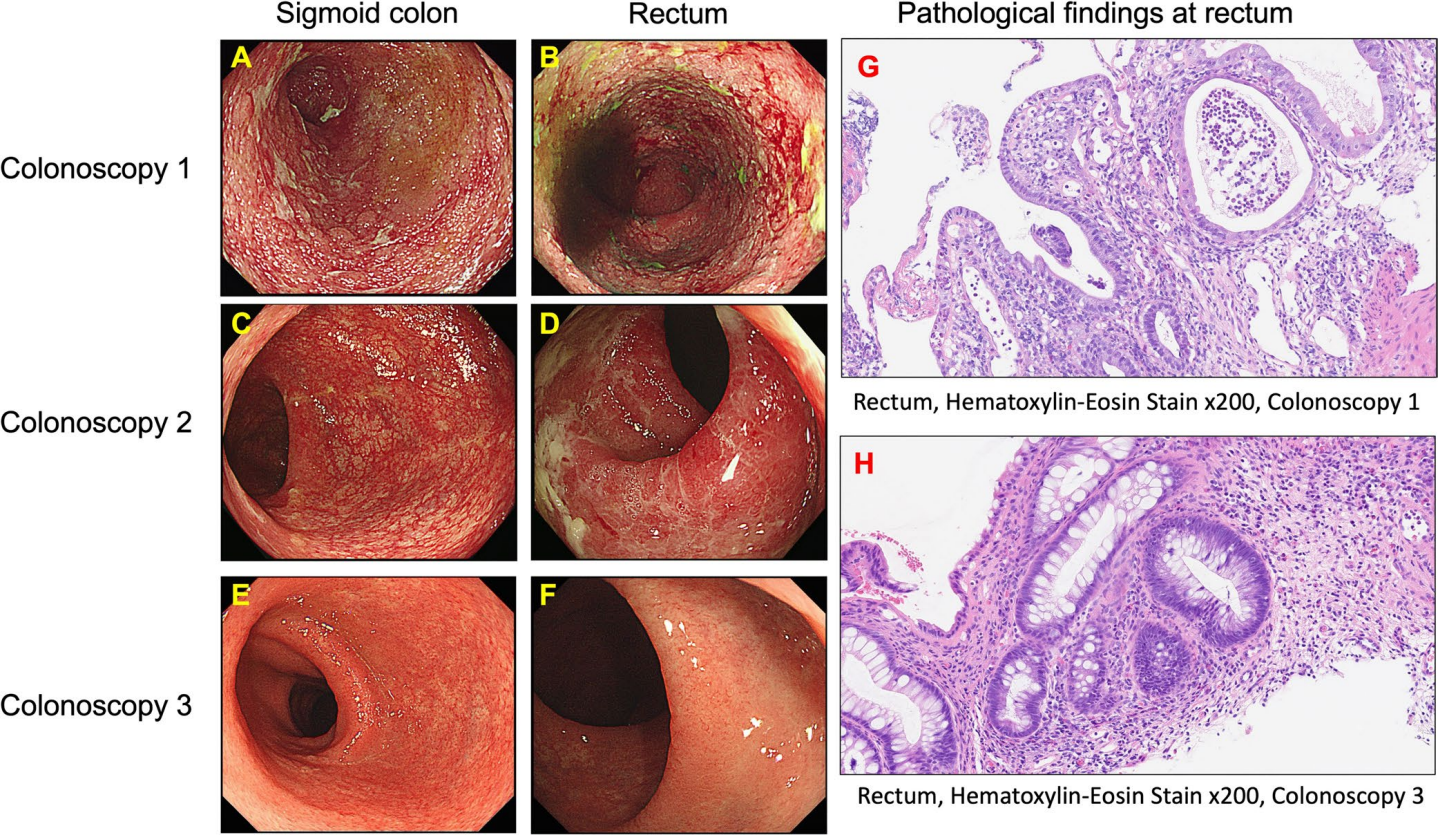
Endoscopic and pathological findings. (A, B) Multiple ulcerations with easy bleeding from the sigmoid colon to the rectum (Mayo endoscopic score (MES) 3). (C, D) Improvement in mucosal inflammation in the sigmoid colon, with moderate inflammation remaining in the rectum (MES 1 and 2, respectively). (E, F) Mucosal healing from the sigmoid colon to the rectum (MES 1). (G) Decreased crypt density and goblet cells, with moderate infiltration of lymphocytes, plasma cells, and neutrophils (Geboes grade of 4.1). (H) Mild infiltration of lymphocytes, plasma cells, and neutrophils (Geboes grade of 3.2).

A 26‐year‐old Japanese male was diagnosed with extensive UC (E3: Montreal classification) 1 year prior and was initially treated with 5‐ASA and prednisolone. However, as the intravenous administration of prednisolone did not lead to any improvement in the patient's clinical symptoms, he was diagnosed with steroid resistance. Consequently, infliximab was initiated at a dose of 5 mg/kg along with 50 mg of azathioprine and 4800 mg of 5‐ASA. With this regimen, the PMS score decreased to 0, clinical remission was achieved, and fecal calprotectin (FC) levels declined to 401.7 mg/kg. Despite the initial response, the frequency of stools and rectal bleeding gradually increased 5 months after clinical remission. His PMS was increased to 7, indicating moderate disease activity at day 0 (6 months after clinical remission). Colonoscopy revealed multiple ulcerations with easy bleeding extending from the sigmoid colon to the rectum (Figure 2A,B), corresponding to a MES of 3. A biopsy of the rectum showed decreased crypt density and goblet cells, along with moderate infiltration of lymphocytes, plasma cells, and neutrophils (Figure 2G), resulting in a Geboes grade of 4.1. Furthermore, his FC level had increased to 3258.7 mg/kg. He was diagnosed with a UC relapse due to a secondary LOR to infliximab. Despite the relapse caused by secondary LOR, he was determined to continue infliximab therapy due to concerns about the limited availability of treatment options in the future. According to the Japanese UC treatment guidelines, GMA is recommended for reducing steroid doses in steroid‐dependent cases [7]. We informed the patient that GMA has also shown promise in managing secondary LOR to infliximab, and he opted to undergo GMA treatment. Consequently, the current treatment, including infliximab, was continued, and GMA was additionally initiated. The GMA induction therapy regimen consisted of 10 sessions, administered twice weekly for 5 consecutive weeks, filtering 1800 mL per session at a rate of 30 mL/min. Heparin was used as the anticoagulant.

Clinical symptoms including abdominal pain, frequency of stools, and rectal bleeding gradually improved, and PMS and FC levels had decreased to 3 and 982.2 mg/kg, respectively, by the end of the 10 sessions. Subsequently, he transitioned to GMA maintenance therapy while continuing infliximab. GMA maintenance therapy was administered every 2 to 4 weeks following the same regimen as induction therapy. By the end of the 4 sessions, his PMS had decreased to 2, indicating clinical remission. After the 14 sessions, his FC level had decreased to 562.2 mg/kg. Colonoscopy at this point showed moderate inflammation remaining in the rectum (Figure 2C,D), and the MES was reduced to 2. Based on these endoscopic findings, GMA maintenance therapy was completed after 15 sessions, and 4 mg of budesonide enema was administered for 90 days. Following the completion of budesonide enema, maintenance therapy with 5‐ASA, azathioprine, and infliximab was resumed, as used prior to the relapse. At 530 days post‐relapse, clinical symptoms had completely resolved, and FC levels had decreased to 374.6 mg/kg. Colonoscopy revealed mucosal healing in the sigmoid colon to rectum (Figure 2E,F), with an MES of 1. A biopsy from the rectum showed mild lymphocyte, plasma cell, and neutrophil infiltration (Figure 2H), with improvement to Geboes grade of 3.2. At 930 days post‐relapse (400 days after completing GMA maintenance therapy), the patient maintained clinical remission while continuing treatment with 5‐ASA, azathioprine, and infliximab.

## 3 Discussion

To our knowledge, this is the first report of GMA induction and maintenance therapy restoring anti‐TNF‐α efficacy and achieving sustained remission in a UC patient with LOR to these agents.

We have previously reported on the efficacy of GMA in patients with active UC and the impact of concomitant pharmacological treatments. Multivariate analysis from 133 cases in that study revealed that the concomitant use of immunomodulators is an independent risk factor for successful GMA remission induction, while LOR to anti‐TNF‐α agents is not a significant risk factor (odds ratio 0.297, *p* = 0.008; odds ratio 0.424, *p* = 0.105, respectively) [21]. Moreover, Yokoyama et al. demonstrated that patients with inflammatory bowel disease (IBD) experiencing LOR to infliximab appeared to regain clinical response to infliximab after undergoing GMA induction therapy, as evidenced by a decrease in the ADA‐to‐infliximab level [22]. However, the long‐term efficacy of sustained remission was unknown in this report because the maximum observation period was 24 weeks. Furthermore, we have reported that GMA induction therapy is also effective for active Crohn's disease with LOR to anti‐TNF‐α agents [26].

Based on these findings, GMA was selected as the induction therapy for the present case. Clinical improvement was observed after 10 sessions of GMA, and the patient was transitioned to GMA maintenance therapy. During GMA maintenance therapy, the patient achieved clinical remission, with a steady decrease in FC levels. However, endoscopic remission had not been achieved by the end of GMA maintenance therapy. After continuing infliximab administration, endoscopic remission was achieved 1 year after completing GMA maintenance therapy. This clinical course strongly suggests that the efficacy of infliximab was restored by GMA maintenance therapy.

Mechanistically, it is known that GMA activates regulatory B‐cells, which produce IL‐10 and exert inhibitory effects on activated lymphocytes. This is thought to be a contributing factor to the prolonged anti‐inflammatory effects observed even after discontinuation of GMA [14, 15, 16]. Moreover, regulatory B cells interact with other immune cells, such as dendritic cells and macrophages, to modulate immune responses. By producing anti‐inflammatory cytokines, regulatory B cells inhibit the maturation and activation of dendritic cells, thereby reducing T cell activation and altering antibody production. Similarly, regulatory B cells suppress macrophage activation, further contributing to the regulation of humoral immunity [27]. Although the exact mechanism by which GMA reduces ADA levels remains unclear, in this case, it is plausible that cytokine modulation by activated regulatory B cells influenced ADA production against infliximab in this case. In other words, it is speculated that prolonged use of GMA, both as induction and maintenance therapy, gradually reduced ADA production and increased infliximab blood concentrations to therapeutic levels, thereby explaining the delayed clinical response observed in this case. Additionally, immunomodulators such as azathioprine are also known to inhibit ADA formation during anti‐TNF‐α therapy [28]. Therefore, it is also conceivable that the concomitant use of immunomodulators with infliximab after GMA completion contributed to the maintenance of long‐term remission by suppressing ADA production low. This likely played a major role in sustaining remission following discontinuation of GMA. However, a critical limitation in this case is that the serum concentration of infliximab was not measured. Therefore, there is no definitive evidence that the effect of infliximab was indeed restored. Monitoring the serum concentration of infliximab may be necessary when treating similar cases in the future.

In conclusion, GMA maintenance therapy might be a new therapeutic option for active UC who have experienced LOR to anti‐TNF‐α agents, restoring the efficacy and allowing for long‐term continuation of such agents.

## Ethics Statement

Written informed consent was obtained from the patient for publication of the case details and accompanying images.

## Conflicts of Interest

The authors declare no conflicts of interest.

## Data Availability

The data that support the findings of this study are available on request from the corresponding author. The data are not publicly available due to privacy or ethical restrictions.
